# Spatiotemporal dynamics of multi-organ pathology and antigen localization in BALB/c mice following intragastric infection with *Brucella melitensis*

**DOI:** 10.3389/fcimb.2026.1907233

**Published:** 2026-10-06

**Authors:** Zhenglong Chai, Chuang Li, Jiarui Luo, Siyu Zhang, Xuanji Zhang, Nannan Pang, Fengbo Zhang, Yuejie Zhu

**Affiliations:** 1 Department of Clinical Laboratory, The First Affiliated Hospital of Xinjiang Medical University, Urumqi, Xinjiang, China; 2 Department of Orthopedic Surgery, The First Affiliated Hospital of Shihezi University, Shihezi, China; 3 Reproductive Medicine Center, The First Affiliated Hospital of Xinjiang Medical University, Urumqi, Xinjiang, China

**Keywords:** *Brucella*, granuloma, immunohistochemistry, intragastric infection, mouse model, pathological dynamics

## Abstract

Brucellosis is a globally distributed zoonosis that invades hosts mainly through the gastrointestinal route, yet the spatiotemporal patterns of organ colonization and tissue injury following intragastric Brucella infection remain poorly defined.

**Objective:**

To investigate the dynamic colonization patterns, histopathological progression, and spatiotemporal antigen localization characteristics of *Brucella melitensis* 16M in multiple murine organs following intragastric infection.

**Methods:**

The mice were randomly divided into an uninfected control group and infection groups euthanized at 1, 3, 7, 14, 28, 42, and 56 days post-infection (dpi). Mice received intragastric inoculation of 10^10^ CFU standard *B. melitensis* 16M strain. Body weight and organ indices were dynamically monitored throughout the experiment. Bacterial loads in the liver, spleen, lung, and mesenteric lymph nodes (MLNs) were quantified by plate spreading assay. Tissue lesions and antigen localization were assessed via hematoxylin and eosin (H&E) staining with semi-quantitative scoring and immunohistochemistry (IHC). Flow cytometry was performed to dynamically quantify total dendritic cells (total DC) alterations in hepatic and splenic tissues at 1, 2, 4, 6, and 8 weeks post-infection.

**Results:**

After intragastric infection, mice developed prominent splenomegaly and transient hepatic weight gain, both peaking at 14 dpi in the acute phase. Splenic bacterial load reached its maximum at 14 dpi, then declined gradually but remained at relatively high levels throughout the chronic phase until 56 dpi. The liver was rapidly colonized in the early stage; hepatic bacterial load rose in the acute phase and decreased markedly in the late chronic stage. Transient infection was observed in the lungs and MLNs. Renal tissue maintained intact histological architecture with consistently negative IHC signals across all time points. Multiple hepatic granulomas were detected, with most IHC-positive antigens confined within granulomatous lesions. In the spleen, severe marginal zone histiocytic infiltration occurred in the acute phase, while an imbalanced red-to-white pulp ratio and abundant megakaryocytes emerged in the chronic phase, with bacterial antigens persistently localized in organized histiocytes. Flow cytometry showed splenic DC proportion peaked at 2 weeks, whereas hepatic DCs increased moderately and plateaued between 2 and 4 weeks.

**Conclusions:**

This study characterizes distinct organ-specific colonization phenotypes, pathological progression, and DC alterations of *Brucella* following gastrointestinal invasion, and clarifies the dynamic between pathogen antigen distribution and local tissue injury. The established dynamic intragastric infection model provides solid morphological evidence for dissecting brucellosis pathological features and developing standardized animal models.

## 1 Introduction

Brucellosis is a global zoonotic infectious disease caused by bacteria of the genus *Brucella* spp. and is recognized by the World Health Organization (WHO) as one of the neglected tropical diseases (Qureshi et al., 2023; Dadar et al., 2025). As a typical Gram-negative intracellular pathogen, *Brucella* has evolved sophisticated immune-evasion mechanisms that enable it to invade and persist within the reticuloendothelial system, particularly macrophages and dendritic cells, thereby establishing chronic infections that are difficult to eradicate (Jiao et al., 2021). Clinically, *Brucella* infection commonly causes nonspecific systemic manifestations, including undulant fever, profuse sweating, arthritis, and hepatosplenomegaly (Jia et al., 2017). In severe cases, it may lead to suppurative lesions in multiple organs and long-term disability, posing a persistent and substantial threat to global public health and the livestock economy (Dadar et al., 2021).

Among the *Brucella* species known to infect humans, *Brucella melitensis* is the most virulent and has the highest pathogenic potential( (Cheng et al., 2026; Li et al., 2026b). In natural settings, the predominant route of infection in humans and animals is ingestion of contaminated dairy products or meat via the digestive tract (Tian et al., 2024). However, most animal-model studies of brucellosis pathogenesis currently employ intraperitoneal (IP) injection or respiratory aerosol infection (Stranahan. Although these non-natural routes facilitate efficient systemic infection, they bypass the host’s first physical and immunological barriers, including gastric acid killing, intestinal mucosal immunity, and the intestinal microbiota, and therefore cannot faithfully recapitulate the initial physiological and pathological processes of oral infection. Accordingly, establishment of an intragastric infection animal model is indispensable for closely simulating the natural route of *Brucella* infection and for clarifying the mechanisms by which the pathogen breaches the intestinal barrier and disseminates to distal organs (Occhialini et al., 2012; Osman et al., 2016; Sánchez-Argáez et al., 2025).

A hallmark pathological feature of *Brucella* infection is inflammatory granulomatous remodeling in target organs, particularly organs of the reticuloendothelial system, such as the liver, spleen, and lymph nodes (Oliveira et al., 2021; Barbieux et al., 2024). Granulomas serve as defensive barriers that restrict pathogen dissemination, yet they may also become intracellular sanctuaries that allow *Brucella* to evade host-specific clearance (Copin et al., 2012). Previous pathological studies have largely focused on single organs or cross-sectional observations at a limited number of time points, lacking long-term tracking of dynamic pathogen colonization across different organs in the whole host (Grilló et al., 2012). Moreover, although pathological injury is closely associated with bacterial burden (Yuan et al., 2026), the spatiotemporal relationship between the specific localization of bacteria in the tissue microenvironment and local organ pathology remains insufficiently characterized. Meanwhile, as central antigen-presenting cells bridging innate and adaptive immunity, the temporal activation and dynamic evolution of dendritic cells (DCs) populations within target organs are also crucial for understanding *Brucella* pathogenicity and host immune responses (Archambaud et al., 2010; Avila-Calderón et al., 2020).

Against this background, the present study established a 56-day long-term intragastric infection model of Brucella melitensis 16M in BALB/c mice. Centering on dynamic phenotypic monitoring of multiple organs, we characterized organ-specific kinetic profiles of bacterial colonization loads, histopathological alterations, spatiotemporal distribution of Brucella antigens, and dynamic variations in the proportion of hepatic and splenic dendritic cells (DCs). This study is intended to complement the dynamic pathological baseline data of gastrointestinal brucellosis and provide a standardized morphological reference model along with clearly defined characteristics of acute and chronic pathological alterations.

## 2 Materials and methods

### 2.1 Experimental animals

The SPF-grade female BALB/c mice required for the experiment, aged 6–8 weeks with a body weight of 18–22 g, were all purchased from the Animal Management Center of Xinjiang Medical University. The mice were acclimated under standard housing conditions for 7 days prior to infection, with the following parameters: temperature 22 ± 2 °C, humidity 50 ± 10 %, 12-hour light-dark cycle, and free access to food and water. All animal experiments complied with the ethical requirements for laboratory animals and were approved by the Medical Ethics Committee of the First Affiliated Hospital of Xinjiang Medical University.

The SPF-grade female BALB/c mice required for the experiment, aged 6–8 weeks with a body weight of 18–22 g, were all purchased from the Animal Management Center of Xinjiang Medical University. The mice were acclimated under standard housing conditions for 7 days prior to infection, with the following parameters: temperature 22 ± 2 °C, humidity 50 ± 10 %, 12-hour light-dark cycle, and free access to food and water. All animal experiments complied with the ethical requirements for laboratory animals and were approved by the Medical Ethics Committee of the First Affiliated Hospital of Xinjiang Medical University.

### 2.2 Bacterial strain and culture

The standard strain *Brucella melitensis* 16M was purchased from the China Institute of Veterinary Drug Control. Bacteria were inoculated onto *Brucella* agar plates (Difco, USA) and cultured at 37°C under 5% CO_2_ for 72 h. Typical colonies were selected and inoculated into *Brucella* broth, followed by shaking culture at 37°C until the logarithmic growth phase. The bacterial suspension was adjusted to OD_600_ = 0.5, corresponding to approximately 1 × 10^8^ CFU/mL, and viable counts were confirmed by plate spreading.

### 2.3 Animal infection

Thirty-five BALB/c mice were randomly assigned to seven post-infection time-point groups: 1, 3, 7, 14, 28, 42, and 56 days post-infection, with five mice in each group. After fasting for 12 h with free access to water, mice were slowly administered 100 µL of *Brucella* suspension by gavage needle, with an infection dose of 10^10^ CFU per mouse (Paixão et al., 2009; Grilló et al., 2012). After gavage, the mouse head was kept tilted upward for 30 s to prevent reflux. Food was withheld for 2 h after gavage, after which free feeding was resumed. Control mice (n = 5) received 100 µL of sterile phosphate-buffered saline (PBS) by gavage. The experimental design is shown in **Figure 1**.

**Figure 1:**
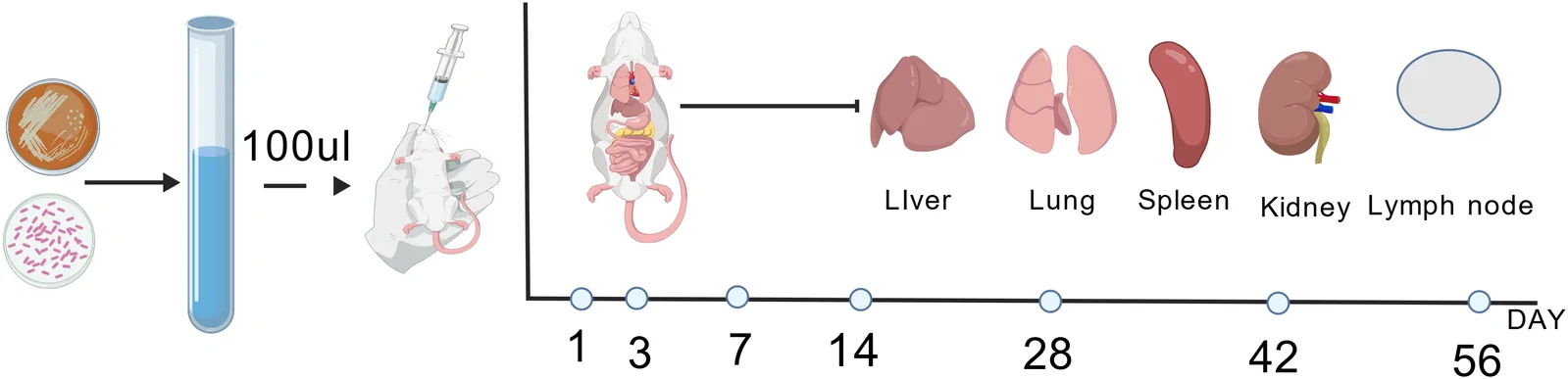
Schematic overview of the mouse model of bacterial infection. Bacterial cultures were prepared and diluted to the appropriate concentration. A volume of 100 μL of the bacterial suspension was injected into mice. Animals were sacrificed at predefined time points (days 1, 3, 7, 14, 28, 42, and 56) post-infection. Including the liver, lung, spleen, kidney, and lymph nodes, were harvested for subsequent analysis. Created with BioGDP.com (Jiang et al., 2025).

### 2.4 Sample collection

In accordance with the experimental schedule, mice were weighed at predetermined time points and subsequently anesthetized via intraperitoneal injection of 3% pentobarbital sodium at a dose of 50 mg/kg. Upon attainment of a stable surgical anesthetic plane, mice were euthanized by CO_2_ inhalation in a dedicated chamber with a continuous gas flow rate of 30–70% of the chamber volume per minute. All euthanasia procedures were strictly performed in compliance with the AVMA Guidelines for the Euthanasia of Animals (Laferriere and Pang, 2020). Complete euthanasia was confirmed by permanent cessation of respiration and heartbeat, followed by cervical dislocation as a secondary physical euthanasia procedure to ensure full mortality. After euthanasia, the liver, spleen, lung, kidney, and mesenteric lymph nodes (MLNs) were aseptically dissected and harvested. All isolated organs were weighed to calculate the organ index, which was determined according to the following formula: Organ index (%) = organ weight (g)/body weight (g)×100. Each organ was then divided into three independent portions for subsequent parallel experiments. For histopathological analysis, tissue samples were fixed in 4% paraformaldehyde at room temperature for 24–48 h. For bacterial load quantification, tissue specimens were homogenized in sterile PBS and immediately subjected to plate counting assays. For flow cytometric detection, the sample size of mesenteric lymph node tissue is limited, and the cell yield is low. Only hepatic and splenic tissues collected at scheduled time points were processed to prepare single-cell suspensions for subsequent immune phenotypic analysis.

### 2.5 Histopathological examination

Paraformaldehyde-fixed tissues were dehydrated through graded ethanol, cleared in xylene, embedded in paraffin, and sectioned at 4 µm. Sections were routinely deparaffinized and rehydrated, followed by hematoxylin and eosin (H&E) staining. All sections were blindly scored by two experienced pathologists who were unaware of the group assignments. The scoring criteria are shown in **Supplementary Tables 2–4**.

### 2.6 Immunohistochemical staining

Paraffin sections were deparaffinized and treated with heat-induced antigen retrieval in citrate buffer (pH 6.0) via microwave irradiation at medium-high power for 10 min. After natural cooling, the sections were incubated with 3% hydrogen peroxide solution for 15 min to block endogenous peroxidase activity. To reduce background staining resulting from non-specific protein binding and block non-specific antigenic sites, tissue sections were completely covered with goat serum blocking solution (ZSGB-BIO, China) and incubated at room temperature for 30–60 min. During blocking, the sections were maintained in a moist environment to prevent desiccation. Subsequently, sections were incubated with a 1:200 diluted anti-Brucella polyclonal antibody (BIOSS, bs-2229R, China) overnight at 4 °C. Following thorough PBS washing, HRP-conjugated goat anti-rabbit IgG (1:500) was applied, and the sections were incubated at 37 °C for 1 h. The sections were visualized with DAB chromogen for 5–10 min, counterstained with hematoxylin, and finally subjected to dehydration, clearing, and mounting. Tissue sections negative for the target antigen were processed and immunostained in parallel with experimental sections to serve as negative controls; the positive control is derived from infected mouse tissues.(For antibody information, please refer to **Supplementary Table 1**).

### 2.7 Determination of bacterial burden

Fresh tissues were weighed, and sterile PBS was added at 1 mL per 0.1 g of tissue. Tissue homogenates were prepared using a sterile homogenizer. Homogenates were subjected to 10-fold serial dilutions from 10^-1^ to 10^-6^, and 100 µL of each dilution was spread onto *Brucella* agar plates. Plates were incubated at 37°C under 5% CO_2_ for 3–5 days. Colonies were counted, and colony-forming units per gram of tissue (CFU/g) were calculated.

### 2.8 Flow cytometry

Mice harvested at 1, 2, 4, 6, and 8 weeks post-infection were subjected to aseptic isolation of hepatic and splenic tissues, and partial tissue specimens were processed for the preparation of single-cell suspensions. For splenic samples, primary cell suspensions were obtained by gentle mechanical grinding and filtration through a 70 μm cell strainer. For hepatic samples, minced tissues were digested in digestive buffer containing collagenase IV and DNase I under constant temperature shaking at 37°C. After sufficient tissue dissociation, the cell suspension was filtered through a 70 μm cell strainer. The cell suspensions derived from both tissues were centrifuged at 300 × g for 5 min at 4 °C. After discarding the supernatant, erythrocyte lysis buffer was added to lyse red blood cells at room temperature, and pre-cooled PBS was added to terminate the lysis reaction. The samples were centrifuged again, and the pellets were washed and resuspended in PBS for subsequent assays. Prior to cell surface staining, single-cell suspensions were pre-incubated with anti-CD16/32 antibody for Fc block at room temperature in the dark to minimize non-specific antibody binding. The cell samples were then stained with a panel of fluorophore-conjugated antibodies and incubated at 4 °C for 30 min in darkness. After incubation, unbound antibodies were removed by PBS washing, and cell pellets were resuspended for on-board detection. According to Guidelines for the use of flow cytometry and cell sorting in immunological studies (third edition) (Cossarizza et al., 2021), dendritic cells (DCs) were identified via a hierarchical gating strategy. Briefly, intact cellular events were first gated to exclude cell debris and impurities. Single-cell populations were further discriminated based on FSC-A/FSC-H parameters to eliminate doublets and cell aggregates. After gating for viable cells using a viability dye to exclude dead cells, CD45^+^ leukocytes were extracted, and lineage-positive cells (including CD3^+^ T cells, CD19^+^ B cells, CD49b^+^ NK cells, and Ly6G^+^ neutrophils) were excluded to obtain the Lin^-^ population. Finally, CD11c^+^MHC-II^+^ cells were gated and defined as total DCs. Flow cytometry data were acquired using a BD FACS Canto II/Fortessa flow cometer. For two-organ flow cytometry analyses, each sample must contain at least 20,000 valid single-cell events to eliminate data bias arising from variations in organ tissue weight, and all data were analyzed using FlowJo software (version 10.8.1). The Circle Gate Strategy is shown in **Supplementary Figure 2**.

### 2.9 Statistical analysis

All data are presented as mean ± standard deviation (Mean ± SD). Statistical analyses were performed using GraphPad Prism 10.1.2. Comparisons among multiple groups were conducted using one-way analysis of variance (ANOVA) followed by Tukey’s multiple-comparison test. Comparisons between two groups were performed using Student’s t-test. Nonparametric data were analyzed using the Kruskal–Wallis test followed by Dunn’s *post hoc* test. *P* < 0.05 was considered statistically significant.

## 3 Results

### 3.1 General condition and dynamic changes in body weight of experimental animals

To clarify the systemic pathological injury caused by *Brucella* infection in BALB/c mice and its temporal characteristics, body weight, major organ weight, and organ indices at each infection time point were analyzed using one-way ANOVA and Dunnett’s *post hoc* test. The results are presented below.

#### 3.1.1 Dynamic changes in body weight of infected mice

The analysis results demonstrated significant differences in mouse body weight across different infection time points (F = 19.044, P <0.001). As shown in **Figure 2A**, during the early phase of infection (days 1–3), mouse body weight exhibited a slight decrease; the mean weight declined slightly from 19.33 ± 0.35 g in the control group to 18.90 ± 0.35 g (P> 0.05). Starting from day 7, the mouse body weight gradually recovered and showed a steady increasing trend. During the middle and late stages of infection (days 42 and 56), mouse body weights reached 21.74 ± 0.36 g and 22.50 ± 1.07 g, respectively, representing significantly higher values compared to the uninfected control group (both P <0.001), which aligns with the natural growth pattern observed in BALB/c mice throughout their postnatal period.

**Figure 2:**
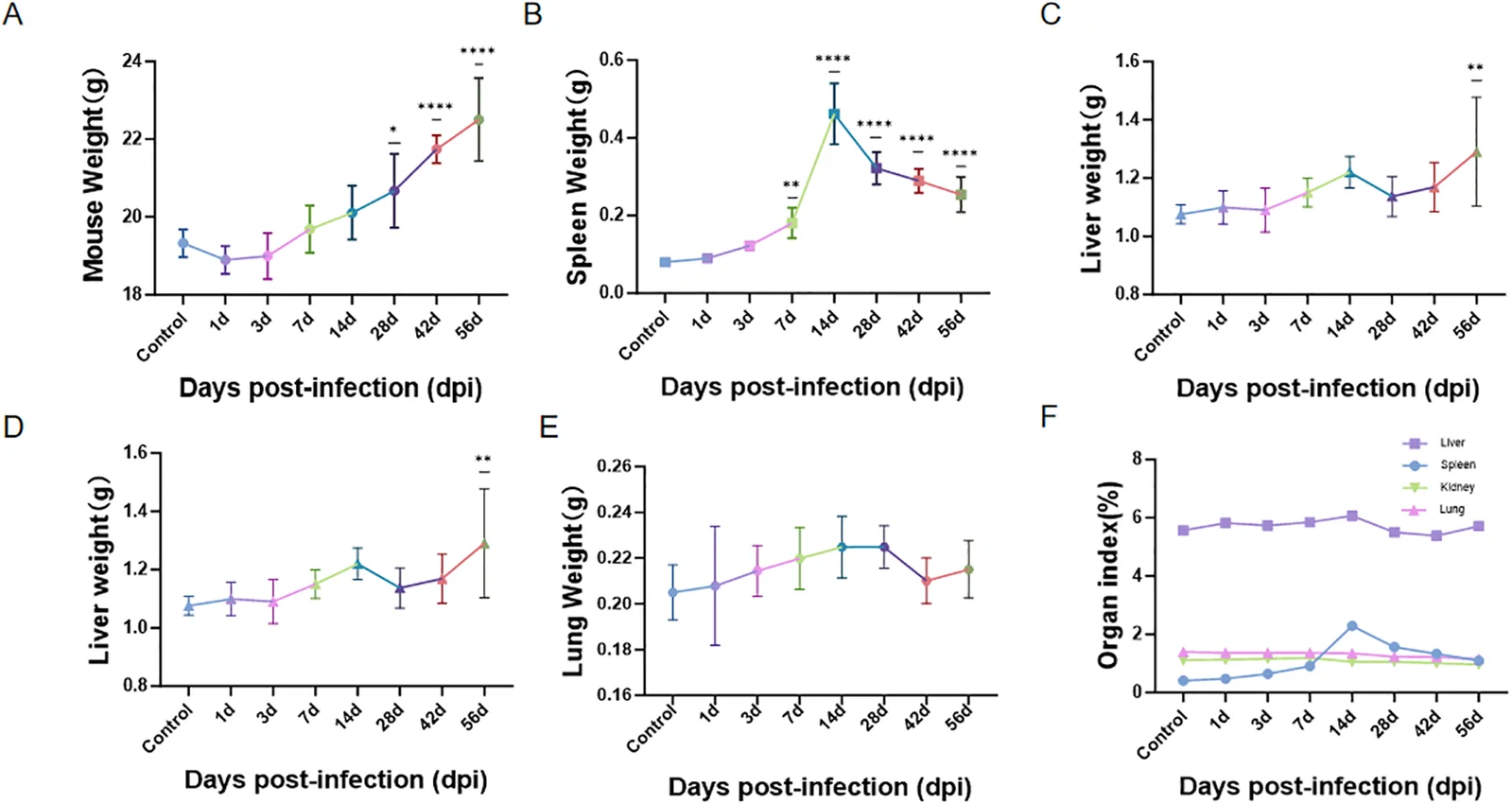
Dynamic changes in body weight, major organ weights, and organ indices after infection. **(A)** Body weight changes at different time points after infection (g). **(B–E)** Absolute weights of the spleen **(B)**, liver **(C)**, kidney **(D)**, and lung **(E)** after infection (g). **(F)** Comprehensive comparison curves of organ indices (%) for the liver, spleen, kidney, and lung. Data are presented as mean ± standard deviation. Compared with the control group, *P < 0.05, **P < 0.01, ***P < 0.001, ****P < 0.0001. Note: organ index (%) = organ weight (g)/body weight (g)×100.

#### 3.1.2 Characteristic splenomegaly induced by *Brucella*

As a critical immune organ, the spleen serves as the primary target organ for Brucella invasion. **Figure 2B** illustrates the dynamic alterations in spleen weight at different time points post-infection. Statistical analysis revealed significant temporal changes in both spleen weight (F = 50.474, P < 0.001) and spleen index (F = 46.257, P < 0.001) throughout the disease course. During the acute ascending phase (1–7 days post-infection, dpi), the spleen index increased slightly from 1 dpi to 3 dpi, with no significant difference compared with the control group (P > 0.05). At 7 dpi, the spleen index rose markedly to 0.9132 ± 0.1785 (P < 0.01). Both spleen weight and spleen index peaked at 14 dpi, representing the peak stage of disease progression. At this time point, the spleen weight reached 0.4617 ± 0.0787 g, and the spleen index increased to 2.2262 ± 0.3429, approximately 5.4-fold higher than that of the control group, with a highly significant statistical difference (P < 0.001). Although splenic hyperplasia gradually declined after the 14 dpi peak, the spleen index remained significantly elevated at 1.1344 ± 0.2331 at 56 dpi (P < 0.001) and failed to return to the normal baseline level.

#### 3.1.3 Response characteristics of the liver, kidney, lung, and other organs

As shown in **Figures 2C**, one-way ANOVA of hepatic weight revealed significant temporal differences in liver weight across different time points (F = 3.326, P = 0.0084), whereas no significant temporal variation was observed in the liver index (P > 0.05). The dynamic changes in hepatic weight were consistent with the alterations in mouse body weight. Post-hoc analysis demonstrated that the liver weight reached its maximum level of 1.220 ± 0.054 g at 14 dpi, which was significantly higher than that in the control group (P < 0.05), and the liver index declined moderately during the late infection stage. As illustrated in **Figures 2D, E**, neither renal weight (P = 0.2239) nor pulmonary weight (P = 0.6836) exhibited statistically significant changes throughout the entire experimental period. Nevertheless, the renal index decreased significantly at the late stage of infection (28 d, 42 d, and 56 d) (all P < 0.05). Collectively, the organ index results in **Figure 2F** indicate that Brucella infection induces a characteristic dynamic pathological progression in mice, predominantly manifested as persistent splenic enlargement and transient hepatic weight elevation.

### 3.2 Dynamic changes in bacterial burden across organs

#### 3.2.1 The spleen is the major bacterial reservoir for *Brucella*

Statistical analysis of splenic bacterial loads demonstrated that tissue bacterial colonization levels were significantly increased at all post-infection time points compared with the control group (**Figure 3A**). Tukey’s multiple comparison test revealed a unique progressive cumulative colonization dynamic of *Brucella* in the mouse spleen. In the early infection stage, the splenic bacterial load exhibited a gradual increasing trend among adjacent time points (1 d vs 3 d, 3 d vs 7 d, and 7 d vs 14 d), whereas pairwise comparisons showed no statistically significant differences (all P > 0.05), indicating a relatively moderate increase in bacterial burden during this period. With the progression of infection and continuous bacterial accumulation, this progressive increment culminated in the peak splenic bacterial load at 14 dpi, representing the maximum bacterial colonization level during the acute infection phase. Following the acute proliferation peak at 14 dpi, the splenic bacterial burden declined slowly over time. Compared with the peak value at 14 dpi, the splenic bacterial load decreased significantly at 56 dpi (14 d vs 56 d, P < 0.01). Notably, in the middle and late chronic stages of infection, pairwise comparisons among adjacent time points (28 d vs 42 d, 42 d vs 56 d, and 28 d vs 56 d) showed no significant differences in residual bacterial loads (all P > 0.05). These results indicated that the splenic bacterial burden tended to stabilize and remained sustained at a relatively constant level during the late infection stage. The dynamic proliferation characteristics and underlying regulatory mechanisms of persistent *Brucella* colonization require further verification.

**Figure 3:**
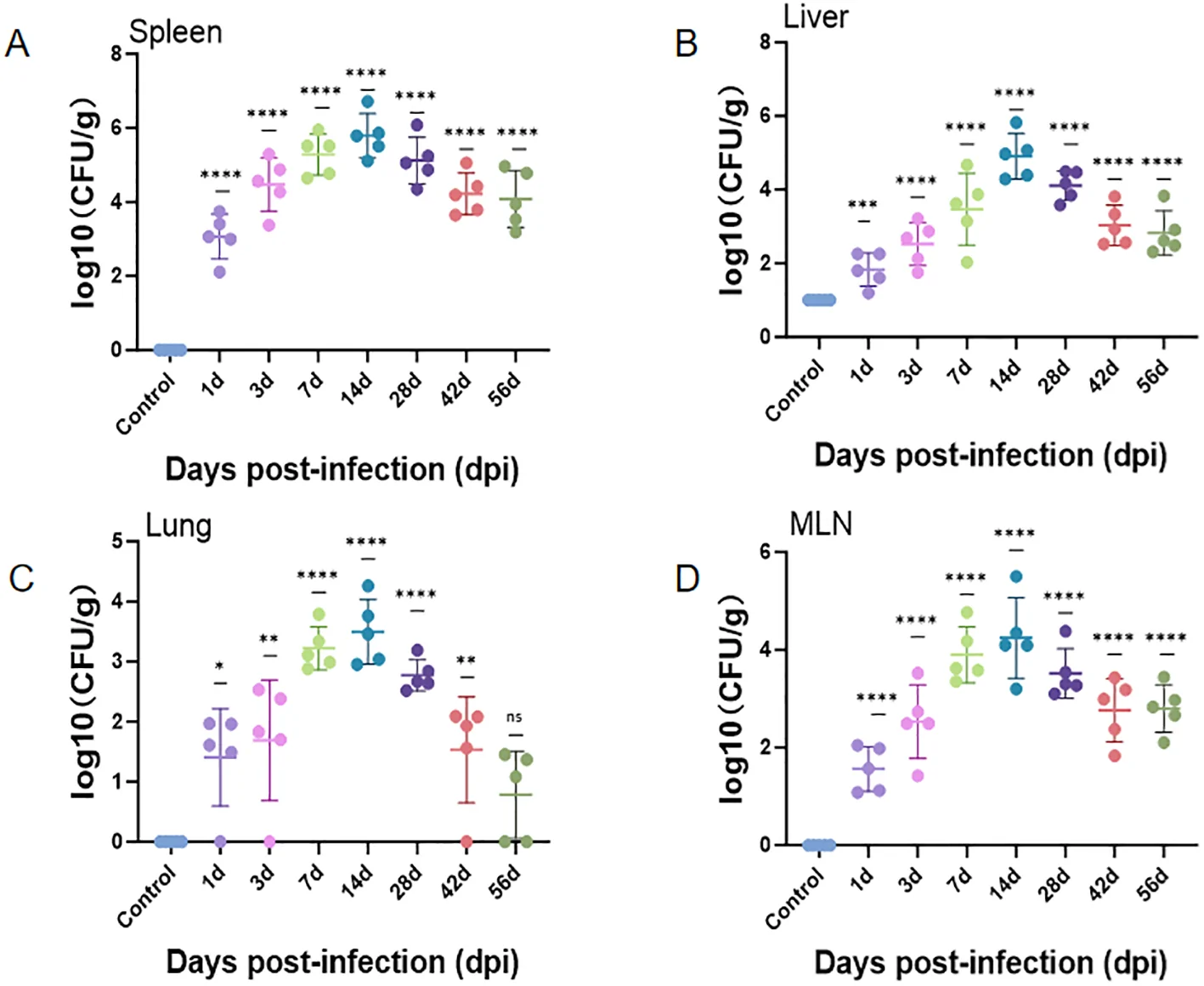
Dynamic changes in bacterial load across various tissues and organs in mice following Brucella infection. **(A)** Spleen; **(B)** Liver; **(C)** Lung; **(D)** Mesenteric lymph nodes. Data are presented as mean ± standard deviation (Mean ± SD) (n = 5 per group). Comparisons were made between the uninfected control group and the infected groups. One-way analysis of variance (One-way ANOVA) was employed for statistical analysis, followed by Tukey’s *post hoc* test to perform pairwise comparisons between different time points during the disease course. Asterisks indicate statistically significant differences between different infection time points (*P <0.05, **P <0.01, ***P <0.001, ****P <0.0001).

#### 3.2.2 Characteristic of *Brucella* bacterial burden in liver tissue

Statistical analysis revealed significant temporal dynamic changes in hepatic bacterial loads following *Brucella* infection in mice (F = 13.53, P < 0.001). Tukey’s post-hoc multiple comparison test was applied for horizontal pairwise analysis among different post-infection time points, which identified distinct hepatic bacterial kinetic characteristics, including rapid bacterial proliferation in the early stage and a moderate decline during the acute phase (**Figure 3B**). In the present study, pathogenic bacteria rapidly established hepatic colonization. *Brucella* was detectable in the liver as early as 1 dpi, followed by a significant increase in bacterial burden that continued to rise until 7 dpi, indicating that the liver serves as a critical organ for early bacterial proliferation and colonization during acute infection. The hepatic bacterial load reached its acute-phase peak at 14 dpi, which was significantly higher than that at the early time points of 1 dpi and 3 dpi (all P < 0.001). A high magnitude of residual bacteria was still maintained at 28 dpi. Compared with the peak level at 14 dpi, the hepatic bacterial burden decreased significantly at 42 dpi (P < 0.01). By the late chronic stage (56 dpi), the hepatic bacterial load declined further and exhibited a continuous downward trend. Other studies have also shown that the liver is capable of clearing residual *Brucella* at approximately 9 weeks (Stranahan et al., 2019).

#### 3.2.3 Characteristics of *Brucella* bacterial burden in the lung

The lung tissue results showed that on days 1 and 3 post-infection, no detectable Brucella colonies were found in some lung samples; however, starting from day 7 post-infection (7 dpi), viable bacteria became consistently detectable in the lung tissue, with the bacterial load initially increasing and then gradually decreasing as the disease progressed. During the acute phase, a peak bacterial load was observed in lung tissue between days 7 and 14 post-infection. Post-hoc multiple comparison tests revealed that the lung bacterial loads at days 7 and 14 were significantly higher than those at the earlier time points of days 1 and 3 (P <0.01 or P <0.05). By day 28 during the mid-to-late infection stage, the bacterial load in the lung tissue decreased by several orders of magnitude. At days 42 and 56, bacteria could no longer be detected in some tissues. As shown in **Figure 3C**, the CFU dynamics exhibited a “slow rise followed by rapid decline” pattern, typically characterized by a sharp decrease occurring 2–4 weeks after the infection reached its peak.

#### 3.3.1 Dynamic characteristics of hepatic histopathology and Brucella antigen localization

In this study, spatiotemporal correlation analysis of sequential tissue sections combining H&E histological staining and anti-*Brucella* immunohistochemistry (IHC) was performed to elucidate the dynamic pathological alterations in the mouse liver within 56 days post-infection (**Figure 4A**, Negative and positive controls are shown in **Supplementary Figure 1**). Global non-parametric statistical analysis confirmed that infection duration exerted a highly significant effect on hepatic pathological lesions (**Figure 4B**, while the individual item scores are shown in **Supplementary Figure 3**). During the initial and progressive stages of infection (1–7 dpi), hepatic parenchymal damage increased gradually, with pathological scores rising progressively from 1.4 at 1 dpi to 2.8 at 3 dpi and 3.5 at 7 dpi. At 7 dpi, H&E staining showed progressive extension of inflammatory cells surrounding the portal areas, and IHC staining revealed that newly formed inflammatory foci began to enclose antigen-positive bacterial clusters. Nevertheless, pairwise comparison indicated that the pathological scores at 1, 3, and 7 dpi exhibited no significant differences compared with the uninfected control group (all scores = 0, P > 0.05). With disease progression, the hepatic pathological damage peaked markedly at 14 dpi, with repeated scoring values ranging from 5.0 to 6.5 (median = 5.5, mean = 5.7), which was significantly higher than that in the control group (P < 0.0001). Microscopically, numerous mature granulomas were widely distributed throughout the hepatic tissue, and abundant *Brucella* antigens were strictly confined within the granuloma cores. During the chronic persistent stage (28–56 dpi), the acute inflammatory response was gradually alleviated. The pathological score slightly decreased to 4.0–6.0 (mean = 4.9) at 28 dpi and subsequently stabilized at a relatively constant level of 4.0–5.0 from 42 dpi to 56 dpi. Although diffuse inflammatory infiltration was attenuated and granulomatous lesions became organized and consolidated during the chronic phase, the pathological scores remained significantly higher than the baseline level of the control group until the experimental endpoint at 56 dpi (P < 0.01). In some samples, positive antigen staining was consistently confined to the granuloma.

**Figure 4:**
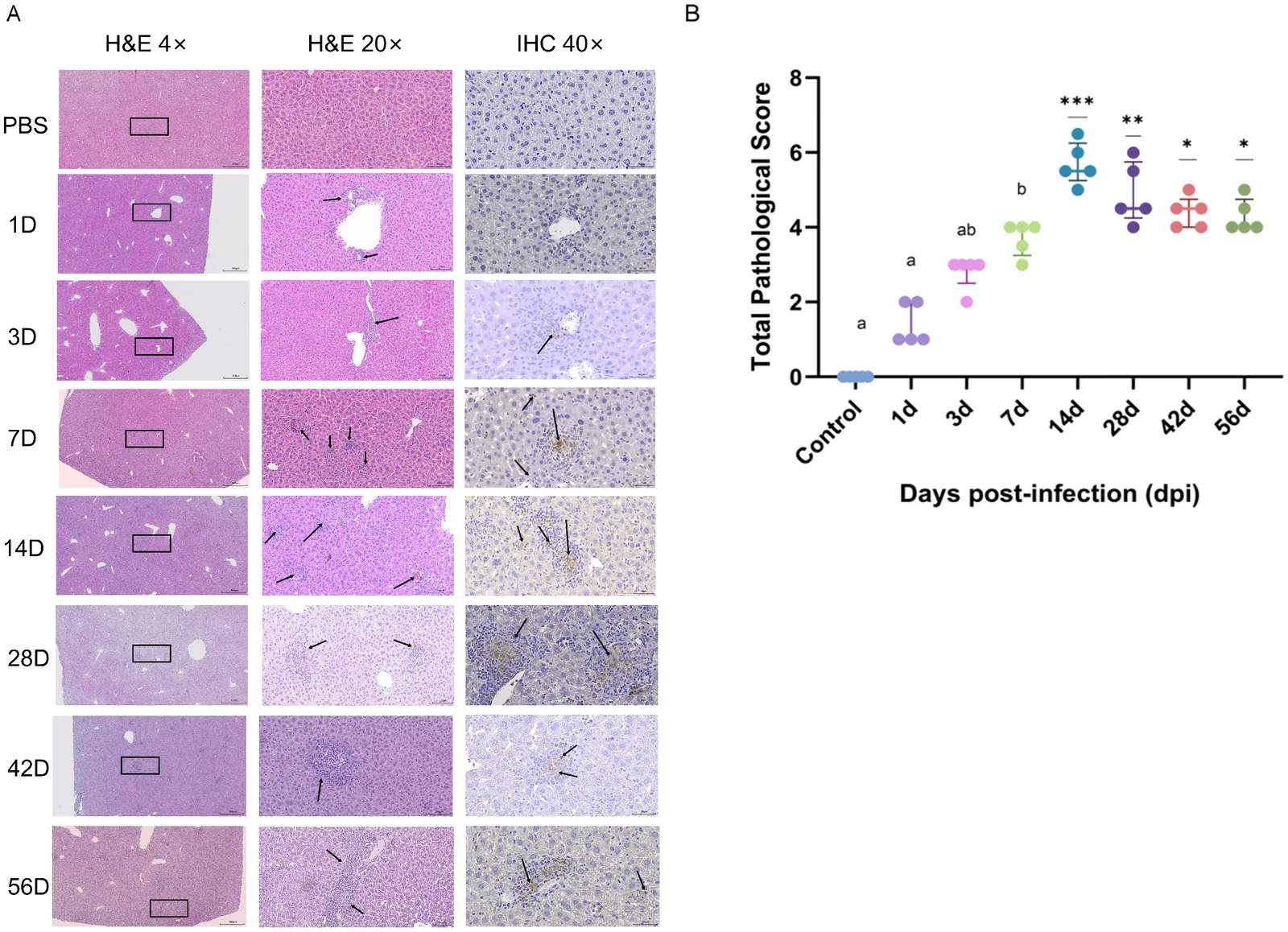
**(A)** Representative images of histopathological and immunohistochemical changes in serial liver sections: representative images of liver sections at each time point are shown. Each time axis consists of three parallel images: the left column shows low-power H&E staining fields (4×; scale bar = 500 μm); the middle column shows medium-to-high-power H&E staining fields (20×), corresponding to magnified regions within the boxed areas in the low-power images, with black arrows indicating granulomas and periportal inflammation (scale bar = 100 μm); the right column shows high-power anti-*Brucella* antigen IHC staining fields (40×), with dark brown specific positive signals indicated by arrows (scale bar = 50 μm). **(B)** Dynamic quantitative scoring of hepatic histopathology: data are presented as median with interquartile range (Median with IQR; n = 5 per group). Two independent statistical multiple comparisons are shown. Lowercase letters (a, b, ab) above data points represent results of independent multiple comparisons within the 0–7 dpi cohort; different letters indicate significant between-group differences (ab *P* < 0.05, b *P*<0.01). Asterisks above data points represent statistical differences from the uninfected control group in global multiple comparisons across the full disease course (0–56 dpi) (*P < 0.05, **P < 0.01, ***P < 0.001), determined by the Kruskal–Wallis test followed by Dunn’s *post hoc* correction. Detailed scoring criteria are provided in **Supplementary Material Table S2**.

#### 3.3.2 Pathological features of the spleen and the spatiotemporal localization of *Brucella* antigen

The results of the semi-quantitative scoring of splenic tissue architecture and immunohistochemical (IHC) antigen localization are as follows(**Figures 5A, B**): At the early stage of infection (1–3 dpi), the overall splenic architecture remained largely intact, with a clear anatomical distinction between the red pulp and white pulp; H&E staining revealed only mild widening of the marginal zone. The pathological scores at 1 dpi (median = 0.0) and 3 dpi (median = 1.0) showed no statistically significant difference compared to the uninfected control group (both scored 0; P> 0.05); corresponding IHC analysis identified the earliest antigen-positive signals in the peripheral regions of the marginal zone. As the disease progressed, by 7 dpi post-infection, the pathological score of the mouse spleen increased significantly (median = 2.0; p <0.05 compared to the Control group), with microscopic examination revealing abundant Brucella antigen signals that transitioned from isolated granular patterns to dense reticular positive bands spanning the entire affected marginal zone. At the peak outbreak phase at 14 dpi post-infection, the semi-quantitative score surged dramatically to its maximum value (median = 4.0; p <0.0001), with microscopic observation showing proliferating cellular infiltrates filling the marginal zone, resulting in blurred boundaries between the white pulp and a pronounced IHC-positive antigen immune response confined within these infiltrated tissue cells. During the intermediate chronic phase (28–56 dpi), unlike the liver, the splenic scores in infected mice consistently remained at elevated levels, with a score of (median = 4.0) at 28 dpi, followed by stabilization at 42 dpi and 56 dpi (all median scores = 4.0; p <0.0001). It is noteworthy that under microscopy, numerous giant cells with large volumes and lobulated nuclei were observed, exhibiting clustered or scattered hyperplasia. At the experimental endpoint of 56 dpi, a strong positive antigenic immune response continued to be detected within the red pulp infiltrates; when combined with the CFU counts, these findings indicate that the spleen serves as the primary site of Brucella colonization.

**Figure 5:**
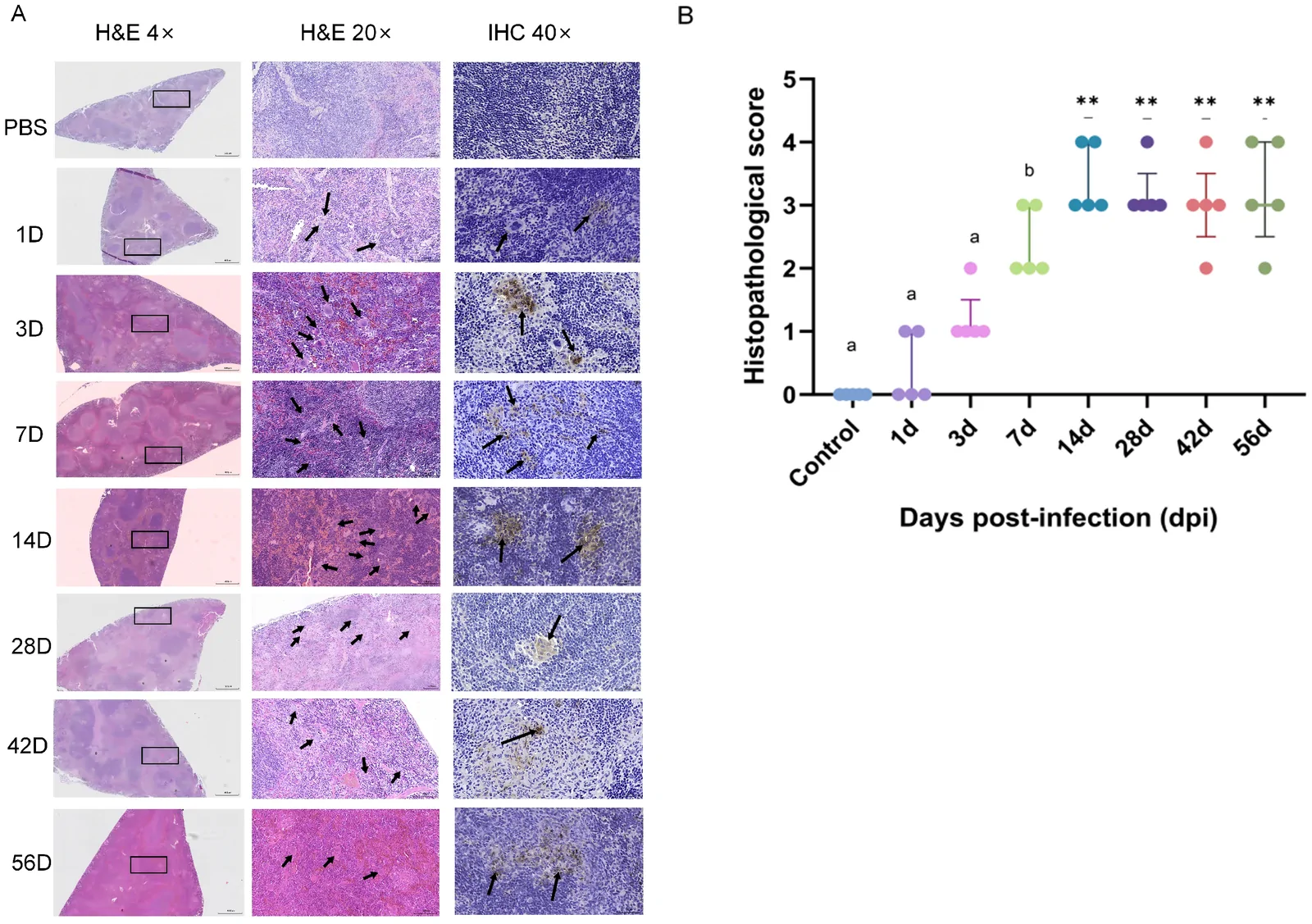
**(A)** Representative images of histopathological and immunohistochemical changes in serial spleen sections: representative microscopic images of spleen sections at each time point are shown. Each time-axis milestone consists of three parallel images: the left column shows low-power H&E staining fields (4×), used to assess the integrity of the red pulp–white pulp boundary, germinal center remodeling, and macroscopic distribution of marginal-zone expansion (* indicates marginal zone; scale bar = 500 μm); the middle column shows medium-to-high-power H&E staining fields (20×), corresponding to magnified boxed areas in the low-power images, highlighting megakaryocytes indicated by arrows (scale bar = 100 μm); the right column shows high-power anti-*Brucella* antigen IHC staining fields (40×), used to precisely define pathogen colonization niches in the splenic reticuloendothelial system, as indicated by arrows (scale bar = 50 μm). **(B)** Dynamic pathological scoring of histiocytic infiltration in the splenic marginal zone: data statistics and plotting were performed using GraphPad Prism. Data are presented as median with interquartile range (Median with IQR; n = 5 mice per group). Two independent statistical multiple comparisons are shown. Lowercase letters (a, b) above data points represent results of independent multiple comparisons within the 0–7 dpi cohort; different letters indicate significant between-group differences (ab *P* < 0.05, b *P*<0.01). Asterisks above data points represent statistical differences from the uninfected control group in global multiple comparisons across the full disease course (0–56 dpi) (**P < 0.01, ****P < 0.0001), determined by the Kruskal–Wallis test followed by Dunn’s *post hoc* correction. Detailed scoring criteria are provided in Supplementary Material **Supplementary Table 3**.

#### 3.3.3 Spatiotemporal relationship between pulmonary alveolar structural remodeling and *Brucella* antigen localization

Subsequently, by coupling microscopic structural evaluation of H&E-stained lung tissue with a quantitative pathological scoring system based on alveolar collapse severity, we analyzed the dynamic patterns of lung injury in mice following Brucella infection. (**Figure 6A**) As demonstrated by the semi-quantitative analysis based on non-parametric tests (**Figure 6B**), during the early stages of infection (1–3 dpi), the morphological structure of the mouse lungs remained relatively intact; the semi-quantitative scores at 1 dpi [median = 0.0 points] and 3 dpi [median = 1.0 points] showed no statistically significant differences compared with the uninfected control group (all scored 0 points; P> 0.05), with most alveolar spaces exhibiting good expansion and uniform size. By 7 dpi post-infection, pulmonary lesions exhibited a progressive worsening trend (median = 2.0 points; p <0.01 vs. Control group), where microscopic examination revealed diffuse hyperplasia and thickening of the alveolar septa in certain regions. At 14 dpi post-infection, the immunopathological damage in the lungs reached its kinetic peak throughout the disease course (median = 2.0 points; p <0.01 vs. Control group), with microscopic observation showing severely thickened alveolar septa accompanied by extensive inflammatory cell infiltration, as well as atrophy and diffuse collapse of most alveolar spaces; at this stage, immunohistochemical staining of serial lung sections revealed deep brown Brucella antigen granules within these collapsed alveolar septa and perivascular areas. During the intermediate chronic phase (28–56 dpi), the lung scores of the mice steadily declined from 28 dpi (median = 2.0 points), reaching significantly lower values at 42 dpi (median = 1.0 points) and at the endpoint of the experiment at 56 dpi (median = 1.0 points). Compared to the Control group (P> 0.05), the IHC antigen signal was attenuated; however, positive signals could still be detected in some cases.

**Figure 6:**
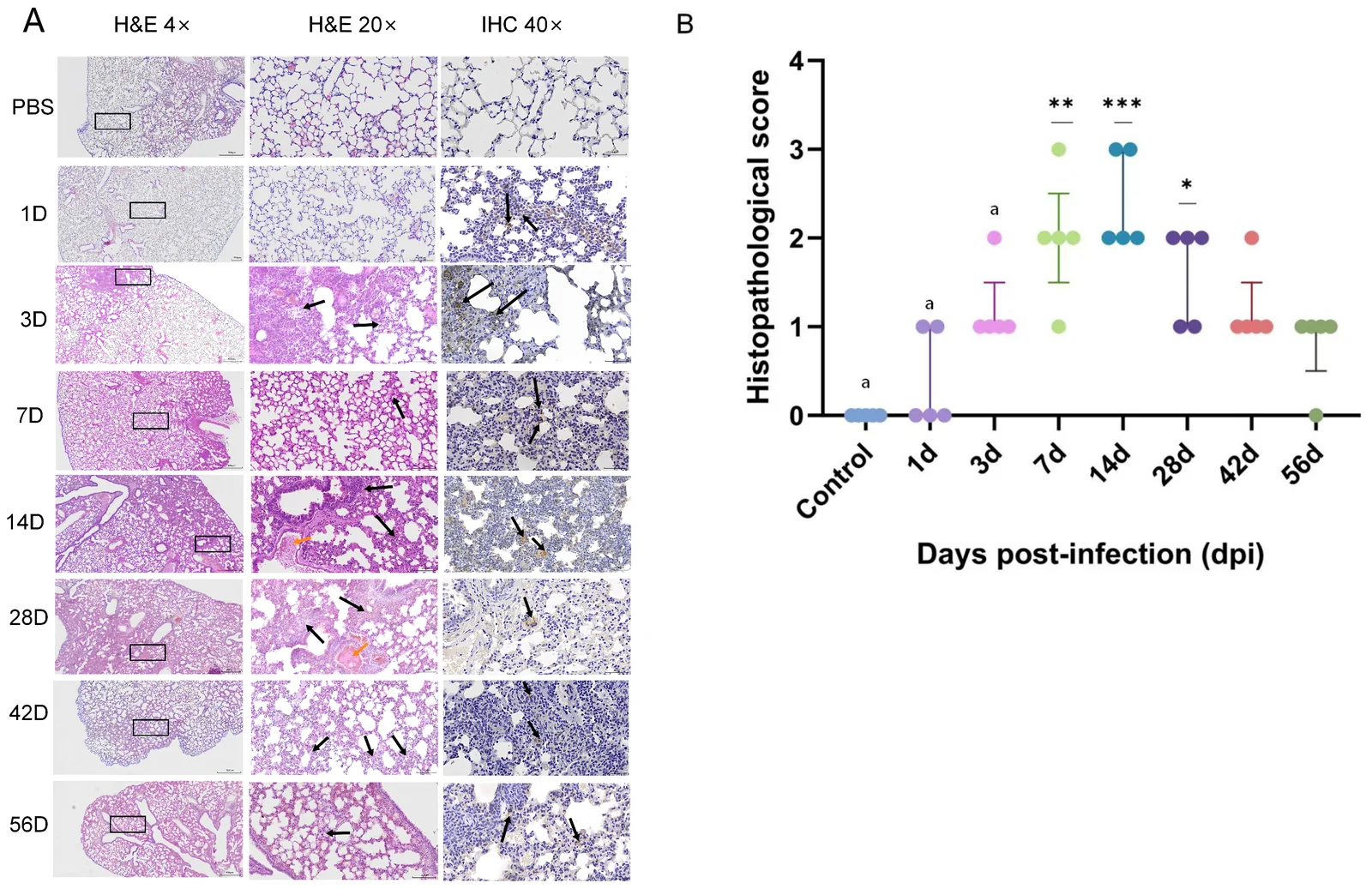
**(A)** Representative images of histopathological and immunohistochemical changes in serial lung sections: representative microscopic images of lung sections at each time point are shown. Each time-axis milestone consists of three parallel images. The left column shows low-power H&E staining fields (4×), used to evaluate global reduction in alveolar air-space area and the macroscopic distribution of parenchymal consolidation lesions (scale bar = 500 μm). The middle column shows medium-to-high-power H&E staining fields (20×), corresponding to magnified boxed areas in the low-power images, highlighting irregular narrowing and collapse of alveolar spaces caused by progressive thickening of alveolar septa, indicated by black arrows, as well as partial intravascular congestion, indicated by orange arrows (scale bar = 100 μm). The right column shows high-power anti-*Brucella* antigen IHC staining fields (40×), used to precisely trace the spatial localization of the pathogen in the pulmonary microcirculation, with dark brown specific antigen-positive particles indicated by arrows (scale bar = 50 μm). **(B)** Dynamic curve of lung pathological scores based on the degree of alveolar collapse: data are presented as median with interquartile range (Median with IQR; n = 5 mice per group). Two independent statistical multiple comparisons are shown. Lowercase letters (a, b) above data points represent results of independent multiple comparisons within the 0–7 dpi cohort.(ab *P* < 0.05, b *P*<0.01).Asterisks above data points represent statistical differences from the uninfected control group in global multiple comparisons across the full disease course (0–56 dpi) (*P < 0.05, **P < 0.01, ***P < 0.001), determined by the Kruskal–Wallis test followed by Dunn’s *post hoc* multiple comparisons. Time points without asterisks indicate no significant difference from the control group. Detailed scoring criteria are provided in Supplementary Material **Supplementary Table 4**.

#### 3.3.4 Mesenteric lymph nodes and kidney

As sentinel organs of intestinal mucosal immune defense, mesenteric lymph nodes (MLNs) exhibit significant lymphofollicular proliferation and histiocytic infiltration during the acute phase of infection (day 14). In the control group, the MLN architecture was basically normal. By the acute outbreak stage, day 14, IHC staining showed that the antigen signal for Brucella dark brown was highly enriched in the cytoplasm of macrophages (**Figure 7A**). During the chronic phase (day 56), a positive IHC signal was observed tending to move inside the lymph node.

**Figure 7:**
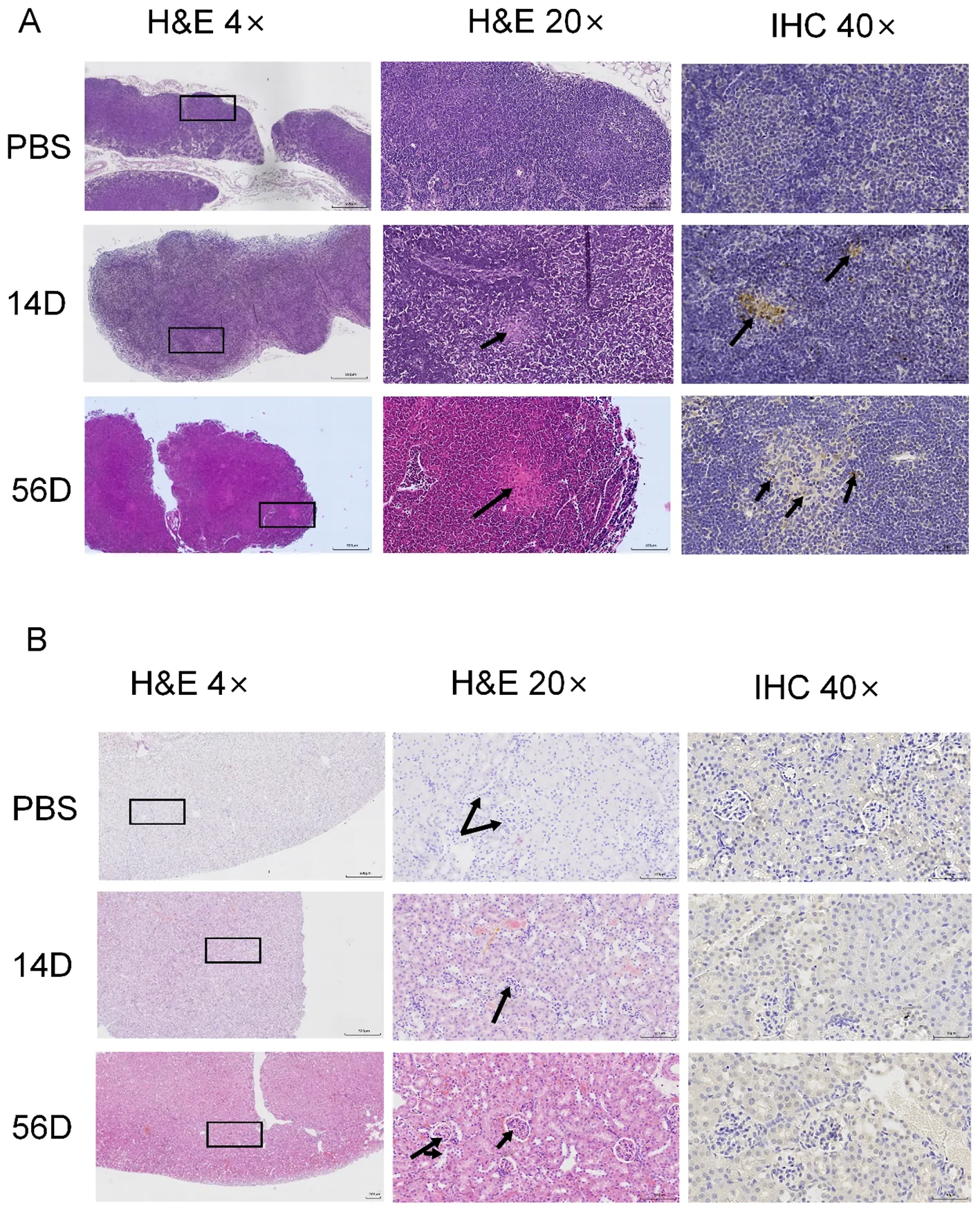
**(A)** Microscopic atlas of serial mesenteric lymph node (MLN) sections (4×, 20×, and 40×): representative morphological changes of MLNs from the acute phase to the chronic phase are shown. Low-power (4×) and medium-power (20×) fields show granulomas indicated by arrows. High-power (40×) IHC staining shows dark brown *Brucella* antigen signals indicated by arrows. **(B)** Microscopic atlas of serial kidney sections (4×, 20×, and 40×): renal tissue architecture during the acute and chronic phases is shown. H&E staining (4× and 20×) shows glomerular and tubular structures in mice at each time point; high-power (40×) IHC staining is shown.

Unlike peripheral organs such as the liver, spleen, and mesenteric lymph nodes, the kidneys are typical non-traditional immune target organs. Assessment was conducted at both the acute phase (14 days) and the chronic phase (56 days). Under the microscope (see **Figure 7B**), the glomeruli and tubule structures of the mice remained highly intact, with no obvious interstitial nephritis, inflammatory cell infiltration, or degeneration and necrosis of tubular epithelial cells. IHC analysis was also performed on continuous sections to confirm that no specific dark brown Brucella antigen-positive signals were detected in the cortex, medulla, or vascular-rich glomerular regions. These morphological features strongly confirm that the kidneys lack sensitivity to Brucella, do not prefer intracellular parasitic niches, and have not undergone significant pathological remodeling during disease progression.

### 3.4 Flow cytometric analysis of dendritic cells in the liver and spleen

Using flow cytometry, we performed a dynamic quantitative analysis of the proportion of total dendritic cells in the spleen and liver of mice at different time points post-brucellosis infection (HC, 1W,2W,4W,6W,8W) (**Figures 8A, B**). The D’Agostino–Pearson test confirmed that the flow cytometric percentage data for both organs followed a standard normal distribution with homogeneous variances. The statistical results (**Figures 8C, D**) demonstrated that, as the disease progressed, the DC-mediated immune response in both organs exhibited distinct temporal patterns. In the spleen, the proportion of DCs among total viable monocytes rapidly increased from baseline levels observed in the healthy control group (HC) at 1W post-infection and reached a dynamic peak throughout the entire disease course at 2W (mean values: 5.79% vs. 0.48%; p <0.0001 compared with the control group), providing robust flow cytometric evidence for the activation of the peripheral immune defense system in the spleen during the acute phase; upon entering the chronic transition phase (4W–8W), as the anatomical structure of the red and white pulp underwent destructive remodeling, the proportion of DCs in the spleen showed a transient slight decline before stabilizing at a plateau level by the experimental endpoint at 8W (mean value: 2.52% ± 0.52%). In contrast to the pronounced surge observed in the spleen, the changes in DCs within the liver were relatively gradual: at 1W and 2W during the acute phase, the proportion of DCs in the liver increased steadily and reached its specific dynamic peak at 2W (p <0.001 compared with the HC group).

**Figure 8:**
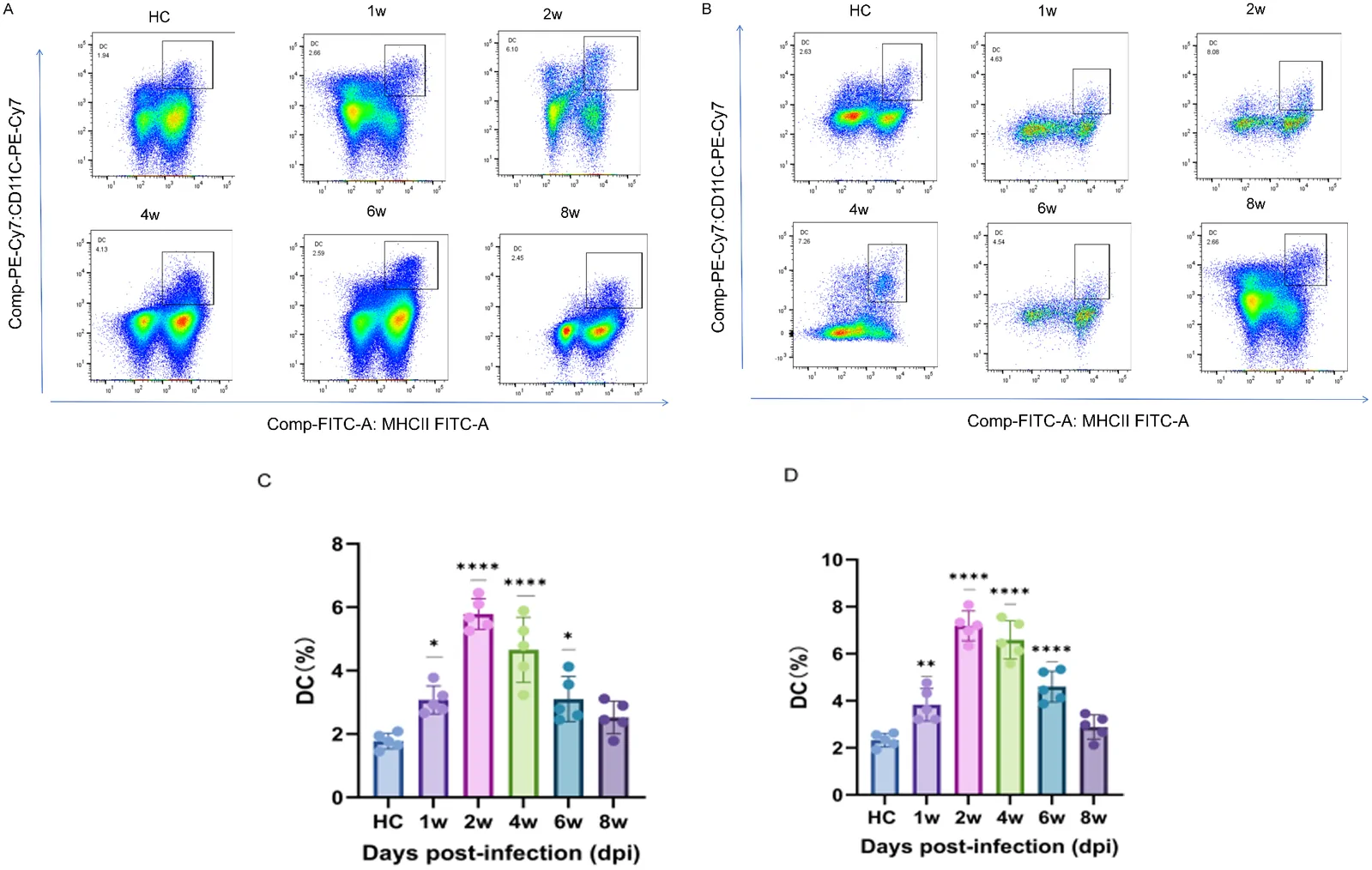
**(A)** Splenic dendritic cells: The left panel shows changes in the proportion of total DCs among total viable mononuclear cells in the spleen at different time points, reflecting the activation level of antigen-presenting function. **(B)** Proportion of hepatic dendritic cells. **(C, D)** Proportions of splenic and hepatic total DCs. All quantitative data are presented as mean ± SD (n = 5 mice per group). Asterisks above data points indicate statistical differences compared with the healthy control (HC) group (*P < 0.05, **P < 0.01, ***P < 0.001, ****P < 0.0001), determined by one-way ANOVA followed by Tukey’s *post hoc* multiple-comparison test. Time points without asterisks indicate no significant difference compared with the HC group (*P* > 0.05).

## 4 Discussion

In this study, BALB/c mice were intragastrically infected with *Brucella melitensis* strain 16M, and the dynamic colonization patterns and histopathological evolution of the pathogen in the liver, spleen, lungs, kidneys, and mesenteric lymph nodes were tracked over 56 days post-infection (dpi), revealing differential organ-specific responses to *Brucella* infection.

The spleen maintained relatively high bacterial loads throughout the entire course. The splenic index, bacterial burden, and H&E pathological scores all peaked at 14 dpi, and during the chronic phase (28–56 dpi), although the bacterial load entered a plateau, the pathological damage scores remained elevated, accompanied by anatomical disorganization of the red and white pulp structures. This finding is consistent with the well-recognized feature of *Brucella* as a facultative intracellular pathogen that preferentially colonizes organs rich in reticuloendothelial cells (Machelart et al., 2017; Willett et al., 2016).

The central role of the spleen in *Brucella* infection is well supported by previous studies (Copin et al., 2012; Hanot Mambres et al., 2015). It has been shown that the majority of primarily infected cells in the mouse spleen are F4/80^+^ myeloid cells, and the peak of infection correlates with granuloma formation, with granulomas composed mainly of CD11b^+^F4/80^+^MHC-II^+^ cells and inflammatory dendritic cells expressing iNOS. Our observation of a low plateau in the proportion of splenic dendritic cells during the chronic phase is consistent with the reported role of dendritic cells as bacterial reservoirs in the chronic stage (Copin et al., 2007).

Of particular note is the mismatch between bacterial load and pathological damage during the late stages of infection. One study revealed that Brucella-induced delayed-type hypersensitivity reactions lead to severe morphological disruption of the spleen at the peak of infection, triggering significant depletion of CD4^+^ and CD8^+^ T cells in the perivascular lymphatic sheaths. This, in turn, causes an imbalance in the ratio of protective lymphocytes to susceptible macrophages, as well as impaired IFN-γ production by splenic cells in response to soluble Brucella antigens. This series of events is not caused by an immune shift from Th1 to Th2 or macrophage-mediated prostaglandin suppression, but stems directly from severe splenic morphological disruption (Hort et al., 2003). Combined with the anatomical imbalance between the red and white pulp observed in this study and the persistently high chronic-phase pathological scores, the extent of chronic tissue damage is partly attributable to ongoing immune-mediated processes, rather than solely to the bacterial load itself (Mora-Cartín et al., 2019).

In the liver, our study revealed a classical pattern of granuloma formation and resolution. Colonization was established as early as 1 dpi, and the peak of dendritic cell immune responses occurred between 2 and 4 weeks. At 56 dpi, although the tissue bacterial burden had decreased, viable bacteria could still be cultured, and positive IHC signals were detectable. Following *Brucella* infection in the liver, macrophages transform into epithelioid cells, forming granulomatous lesions (Daggett et al., 2020). IL-12 plays a critical role in granuloma formation and containment of the pathogen. In the acute phase, the liver exhibits granulomatous inflammatory aggregates, while in the chronic phase, the inflammatory response shifts predominantly to lymphocytic infiltration (Hielpos et al., 2017). The dynamic evolution of granulomas observed in our study is consistent with this transition of immune responses between acute and chronic stages.

In the lungs, we observed an acute-phase rise and chronic-phase decline in bacterial loads. In some animals, early alveolar septal thickening and collapse were noted, but these changes gradually resolved after 28 dpi, although most lungs still showed positive antigen staining. This result is consistent with a recent oral infection study (Li et al., 2026a), which reported that pulmonary and lymph node bacterial loads peaked at 1 week and declined thereafter, yet remained detectable until 8 weeks, with significant resolution of inflammation after the acute phase. Following respiratory infection (Ferrero et al., 2020; Henning et al., 2012), *Brucella* disseminates from the lungs to systemic sites, but in the chronic phase, it primarily colonizes organs such as the spleen. Our histological evidence that the lungs are not the main organ for chronic persistence complements this conclusion.

The present study adopted the intragastric infection route, which differs from intranasal inoculation that directly establishes pulmonary infection (Hielpos et al., 2017; Ferrero et al., 2020). Following gastrointestinal invasion, *Brucella* disseminates to distant organs sequentially via translocation across the intestinal mucosa, transport through mesenteric lymph nodes, and systemic circulation, leading to an obvious time lag for bacterial colonization in the lung (Grilló et al., 2012). Furthermore, the lung is not a preferred reticuloendothelial target organ for *Brucella*. Local pulmonary innate immunity efficiently eliminates trace invading bacteria, rendering viable bacterial loads below the limit of detection in most samples. In addition, oral infection is accompanied by inherent inter-individual variation. Heterogeneity in intestinal absorption efficiency and the extent of bacterial entry into the bloodstream further explains the absence of cultivable bacteria in partial lung tissues during early infection. Accordingly, negative bacterial load results in the lung at early time points merely indicate that viable bacteria fail to reach the detection threshold of culture assays, rather than a complete lack of bacterial invasion. This observation is consistent with the sporadic positive antigen signals detected by IHC in this study.

Theoretical research on brucellosis vaccines is relatively extensive (Chai et al., 2025; Li et al., 2025; Luo et al., 2025). This infection model can more accurately reflect the infection and immune response processes at the mucosal interface, making it particularly suitable for evaluating the immunoprotective efficacy of oral vaccines and mucosal adjuvanted vaccines (Tian et al., 2024; Shang et al., 2025); additionally, it provides a standardized reference benchmark for defining the core endpoint in vaccine protective efficacy assessments (specifically, the degree of reduction in bacterial load in target organs). However, mouse models exhibit certain differences from natural hosts and humans in terms of species-specific immune characteristics and pathological outcomes; therefore, this model is primarily employed for the early screening and mechanistic exploration of vaccine candidates, while the ultimate protective efficacy of such vaccines still requires further validation using larger animal models.

Compared with previous studies, our use of the intragastric route more closely mimics the natural route of infection in humans via the digestive tract (Osman et al., 2019). Although the bacterial loads were lower than those achieved by intraperitoneal injection (Stranahan et al., 2019; Hensel et al., 2020; 2022), the pathological damage patterns were similar, indicating that the intragastric model can effectively reproduce the core pathological features of brucellosis. Furthermore, we employed eight time points and synchronized multi-organ tracking, providing systematic data on the pathological evolution across multiple organs over 56 days. A recent oral infection study with an 8-week observation period is comparable to our design, and the trends in splenic and pulmonary bacterial loads were largely consistent between the two (Li et al., 2026a). Additionally, by combining IHC antigen localization with H&E pathological evaluation, we described the spatial distribution of bacteria in relation to tissue damage.

Several limitations of this study should be acknowledged. First, we used only the virulent 16M strain and BALB/c mice, without including comparisons across different host genetic backgrounds or pathogen virulence variants, and there may be sex-related biases. BALB/c mice are relatively susceptible to *Brucella*, and immune responses in other mouse strains could differ, potentially affecting pathological outcomes. Second, the driving mechanisms underlying the pathological phenotypes require further verification using molecular approaches such as transcriptomics or cytokine microenvironment dynamics (e.g.pro-/anti-inflammatory balance). Third, although the 56-day observation period covers the acute-to-chronic transition, longer-term outcomes (e.g., 180 days) were not tracked. In addition, this study focused mainly on immune organs such as the spleen and liver, while other important target organs of *Brucella*, including the intestine and reproductive organs, were not systematically examined, and future studies could expand on this basis. Last, this study focused on the dynamic changes of total DCs, and further studies with additional markers are required to dissect the roles of distinct DC subsets during Brucella infection (Tian et al., 2026).

In summary, this study utilized an intragastric mouse infection model to enhance the repertoire of available infection models for Brucella. It delineated the temporal dynamics of multi-organ damage following natural-route infection and clearly defined the distinct pathological features of the spleen as the core target organ during both acute and chronic stages. Furthermore, it described the classical evolution of hepatic granulomas, the transient colonization of the lungs, and the kidneys’ non-susceptibility. The integrated evaluation system, which combines IHC bacterial localization with H&E pathological assessment, provides a standardized baseline for animal models that closely mimic the natural infection state. This may facilitate future investigations into Brucella’s immune evasion mechanisms, the evaluation of the protective efficacy of novel mucosal vaccines, and the development of antimicrobial agents targeting intracellular pathogens.

## Data Availability

The original contributions presented in the study are included in the article/**Supplementary Material**. Further inquiries can be directed to the corresponding authors.
