# Supplementary material for: Spatiotemporal dynamics of multi-organ pathology and antigen localization in BALB/c mice following intragastric infection with Brucella melitensis
Source: Front Cell Infect Microbiol. 2026 Oct 6;16:1907233. doi: 10.3389/fcimb.2026.1907233 (PMC13680254; doi:10.3389/fcimb.2026.1907233)
Supplement: Supplementary file 1 [file SupplementaryFile1.docx]

Supplementary Material

Table S1 Antibody Information

| **Product Name** | **Cat No** | **Clon** | **Size** | **Manufacturer** | **Fluorochrome** |
| --- | --- | --- | --- | --- | --- |
| Anti-mouse CD45 Antibody | 557659 | 30-F11 | 0.1mg | BD Biosciences | APC-Cy7 |
| Anti-Mouse CD11c | 558079 | HL3 | 0.1mg | BD Biosciences | PE-Cy7 |
| Anti-Mouse MHC-Ⅱ | 562009 | 2G9 | 50ug | BD Biosciences | FITC |
| Fixable Viability Stain 450 | 562247 | / | 0.1mg | BD Horizon | BV421 |
| Anti-Mouse CD16/CD32 | 553142 | 2.4G2 | 0.1mg | BD Pharmingen | / |
| Brucella Rabbit pAb | bs-2229R | / | 100ug | Bioss antibodies | / |

**Table S2. Semi-quantitative histopathological scoring criteria for murine liver lesions.**

| **Lesion Type** | **Score** | **Description** |
| --- | --- | --- |
| **Periportal inflammation** | 0 | None |
|  | 1 | Minimal - <25% of periportal areas affected |
|  | 2 | Mild - 25% to 50% of periportal areas affected |
|  | 3 | Moderate - 51% to 75% of periportal areas affected |
|  | 4 | Marked - >75% of periportal areas affected |
| **granulomas** | 0 | None |
|  | 1 | Minimal - 1 focus per ten 10× objective fields |
|  | 2 | Mild - 2 to 4 foci per ten 10× objective fields |
|  | 3 | Moderate - 5 to 10 foci per ten 10× objective fields |
|  | 4 | Marked - >10 foci per ten 10× objective fields |

Table S3 Histopathological grading scale for histiocytic infiltration in splenic marginal zones.

| ****Score**** | ****Detailed Pathological Description**** | ****Affected Area**** |
| --- | --- | --- |
| **0** | No histiocytic infiltration observed. The tissue structure is intact, and the marginal zone is clear of inflammatory cells. | 0% |
| **1** | Sparse and scattered infiltration of histiocytes. Inflammatory cells are limited to isolated foci, occupying less than 25% of the target marginal zone area. | < 25% |
| **2** | Noticeable focal infiltration of histiocytes. Inflammatory cells form loose aggregates or clusters within the marginal zone, covering 25% to 50% of the area. | 25% – 50% |
| **3** | Significant histiocytic accumulation. The inflammatory infiltration is coalescing, showing clear evidence of tissue displacement and architectural disruption, covering 51% to 75% of the marginal zone. | 51% – 75% |
| **4** | Extensive and confluent histiocytic infiltration. The marginal zone is densely packed with inflammatory cells, leading to total obliteration of the original tissue structure, exceeding 75% of the area. | > 75% |

Table S4. Semi-Quantitative Histopathological Grading Scales for Pulmonary Lesions

| ****Score**** | ****Alveolar Size & Morphology Criteria**** | ****Affected Area**** |
| --- | --- | --- |
| **0** | **Normal lung tissue.** Alveolar spaces are fully expanded and uniform in size, with no compression or collapse; alveolar septa are thin. | 0% |
| **1** | **Mild reduction / Focal compression.** Mild thickening of alveolar septa (mostly perivascular), causing slight reduction or mild compression of a minority of surrounding alveolar spaces; most alveoli remain normal in size. | < 25% |
| **2** | **Moderate reduction / Partial collapse.** Progressive thickening of alveolar septa with inflammatory cell infiltration, leading to marked narrowing, constriction, or irregular collapse of alveolar spaces in a moderate area; the area of eosinophilic parenchyma approaches that of the clear alveolar spaces. | 25% – 50% |
| **3** | **Severe reduction / Extensive collapse.** Severe thickening and fusion of alveolar septa, leading to drastic shrinkage, severe collapse, or obliteration of the vast majority of alveolar spaces; microscopic examination shows large areas of "consolidation-like" remodeling, with only a few residual micro-airspaces. | 51% – 75% |
| **4** | **Extreme / Global consolidation (Airspace disappearance).** Widespread destruction, fusion, or complete disappearance of alveolar structure across the entire field, with extreme density of inflammatory cell infiltration, resulting in no identifiable alveolar spaces (consolidation rate > 75%) and a "hepatization" state where no ventilation is possible. | > 75% |

**Figure S1.** Specificity validation of the immunohistochemical staining system. **A:** Liver tissue; **B**: Spleen tissue; **C:** Lung tissue. For each organ, the left panel displays the tissue negative control prepared from corresponding tissues of uninfected normal mice, with no specific positive staining observed. The right panel displays the positive control prepared from corresponding tissues of mice at the late stage of Brucella infection with high bacterial load, showing distinct brown immunopositive signals. Nuclei were counterstained with hematoxylin.

**Figure S2:** Flow cytometry gating strategy for mouse tissue-derived dendritic cells (using spleen as an example). **A:** The total cell population is defined based on forward scatter area (FSC-A) and side scatter area (SSC-A); **B:** Cell adhesion complexes are excluded using the forward scatter height (FSC-H) versus FSC-A scatter plot, thereby retaining a single cell population; **C:** The live cell population is identified by gating based on staining with live/dead dyes for subsequent analysis; **D:** CD45-positive cells are gated within the live cell population; **E:** Lineage-negative cells are identified to exclude non-target lineage cells such as T cells, B cells, NK and granulocytes; **F:** Among the Lin⁻ cell population, dendritic cells are finally identified based on the expression of CD11c and MHC-II; **G:** Negative control for total dendritic cell gating.

****Figure S3**.** Semi-quantitative histopathological evaluation of mouse liver tissues. **A:** Semi-quantitative score of portal inflammation; **B:** Semi-quantitative score of hepatic granulomas. All tissue sections were independently evaluated in a blinded manner, with higher scores indicating more severe pathological lesions.
